# Comparison of Anterior and Posterior Approaches for Acute Traumatic Central Spinal Cord Syndrome with Multilevel Cervical Canal Stenosis without Cervical Fracture or Dislocation

**DOI:** 10.1155/2022/5132134

**Published:** 2022-02-16

**Authors:** Quan Zhou, Junxin Zhang, Hao Liu, Xinfeng Zhou, Wei He, Zheyu Jin, Huilin Yang, Tao Liu

**Affiliations:** Department of Orthopaedics, The First Affiliated Hospital of Soochow University, Suzhou, Jiangsu 215006, China

## Abstract

**Introduction:**

This is a retrospective comparative study that aims to compare the benefits of different surgical approaches for patients with multilevel cervical canal stenosis (CCS) without cervical fracture or dislocation of acute traumatic central cord syndrome (ATCCS).

**Methods:**

From January 2015 to December 2018, 59 patients were included in the study. Among them, 35 patients (Group A) received anterior surgery and 24 patients (Group B) received posterior surgery. Primary outcome measures were American Spinal Cord Injury Association (Asia) grade, Japanese Orthopaedic Association (JOA) score, and recovery rate (RR). Secondary outcome measures included operation time, intraoperative blood loss, visual analogue scale (VAS) score, cervical sagittal parameters, and complications. Multivariate linear regression was used to analyze prognostic determinants.

**Results:**

Compared with Group B, Group A had longer operation time and more intraoperative blood loss (*P* < 0.05). However, the VAS score of Group B was higher than that of Group A at discharge (*P* < 0.05). There was no significant difference in cervical sagittal plane parameters between the two groups (*P* > 0.05). Postoperative complications were different in the two groups. During follow-up, the Asia grade, the JOA score, and RR of both groups improved (*P* < 0.05), but there were no significant differences between the two groups (*P* > 0.05). Younger age, earlier surgery, and better preoperative Asia grade were correlated with better prognosis.

**Conclusions:**

For patients with multilevel CCS without cervical fracture or dislocation of ATCCS, both surgical approaches had good outcomes. Although no significant differences were found in the primary outcome measures between the two groups, there were different recommendations for the secondary outcome measures. Younger age, earlier surgery, and better preoperative Asia grade were protective factors for better prognosis.

## 1. Introduction

The incidence of cervical spinal cord injury (SCI) due to acute trauma has been increasing year by year in the world [1], and one of the most common symptoms is acute traumatic central cord syndrome (ATCCS), which accounts for approximately 70% of all incomplete SCI [2–5]. This symptom was first proposed by Schneider in 1954, and its clinical manifestations are as follows: differential weakness of the upper and lower extremities and variable involvement of the sensory system and a variable impact on bladder function [6, 7]. Previous studies have shown that because hyperextension injury exacerbates loss of spinal volume, people with cervical canal stenosis (CCS) before injury are more likely to develop ATCCS than those with normal cervical canal [8–10]. In addition, due to the rapid increase in aging in the world, the number of elderly people with CCS is also increasing [11, 12]. As a result, due to the existing CCS, more and more elderly people are exposed to the risk of ATCCS, which prompts us to conduct more in-depth researches on the treatment of these patients.

Currently, the standard treatment for patients with ATCCS remains unclear. On the one hand, the question of whether and when to perform surgery has always been a matter of debate. An increasing number of studies have shown that early surgical intervention can be performed if persistent compression factors exist after the occurrence of ATCCS [13–15]. On the other hand, the optimal choice of surgical approaches is also controversial. Due to the lack of specific criteria, it is based mainly on the personal experience of spinal surgeons. Anterior approaches included mainly anterior cervical discectomy and fusion (ACDF) and anterior cervical corpectomy, and fusion (ACCF). Posterior approaches included mainly laminectomy and laminoplasty. Current evidence (class III) suggests that there was no significant difference in neurological function between the two anterior approaches, nor was there a significant difference between the two posterior approaches [16]. For patients with preexisting multilevel CCS of ATCCS, the anterior or posterior approach seems be more difficult to choose. Surgeons should choose the appropriate approach to decompress the spinal cord and pay close attention to try to restore the volume of the stenotic spinal canal. Furthermore, when these patients also have ossification of the posterior longitudinal ligament (OPLL), the anterior approach is usually contraindicated due to the presence of adherences between the dura and the ossified posterior ligament, with a high risk of central spinal fluid leak [17, 18].

In this study, we conducted a retrospective comparative study to compare the benefits of anterior and posterior surgical treatments for patients with multilevel CCS without cervical fracture or dislocation of ATCCS. In addition, we tried to analyze the factors that determine the prognosis of these patients.

## 2. Methods

### 2.1. Selection Criteria

Inclusion criteria were as follows: (1) central cord syndrome; (2) developmental CCS whose diameter of the canal is less than 12 mm in two segments or above; (3) history of acute trauma; (4) patients who received shock therapy with methylprednisolone within eight hours after injury.

Exclusion criteria were as follows: (1) the Asia Impairment Scale grade A or E; (2) cervical fracture or dislocation; (3) another type of serious injury occurred at the same time, such as brain injuries, damage to major organs, or other spinal injuries; (4) patients who died or were unable to complete 24 months of follow-up.

### 2.2. General Information

According to the inclusion and exclusion criteria, a total of 59 patients with multilevel CCS without cervical fracture or dislocation of ATCCS were admitted to the same designated hospital from January 2015 to December 2018. Among them, 35 cases (Group A) received ACDF or ACCF and 24 cases (Group B) received posterior laminoplasty or laminectomy. All patients received cervical spine X-ray, computed tomography (CT), and magnetic resonance imaging (MRI) at admission and received cervical spine X-ray after surgery. The basic data of patients in the two groups, including age, gender composition, causes of injury, the narrowest segment and degree of CCS, canal compression rate (CCR), time after injury until operation, time after operation until discharge, the proportion of OPLL, length of stay (LOS), comorbidities and follow-up period, were all recorded.

### 2.3. Treatment Strategy

All patients were hospitalized in our hospital after trauma and received a high dose of MP within eight hours of injury. After shock therapy, spinal surgeons carefully evaluated the patients' imaging results and clinical manifestations and then made recommendations to patients and their families on whether or not to perform surgery and what type of surgery to perform depending on the progression of the patients' diseases. Surgical indications were mainly persistent compression of the spinal cord that correlated with the level of neurological deficit.

**Figure 1:**
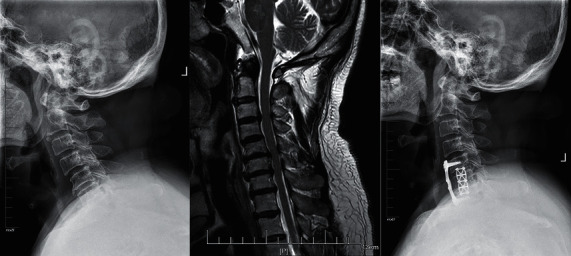
A 44-year-old man who developed symptoms of ATCCS after a car accident was treated with ACCF.

**Figure 2:**
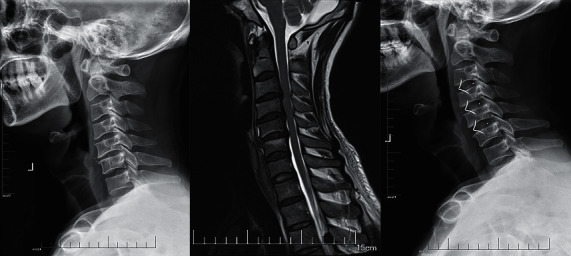
A 47-year-old man who developed symptoms of ATCCS after a fall was treated with ACDF.

In Group A, 35 patients received anterior surgery after injury. Of the 11 patients who underwent ACCF (Figure 1), 5 cases received one-level surgery, 4 cases received two-level surgery, and 2 cases received three-level surgery. Of the 24 patients who underwent ACDF (Figure 2), 11 cases received one-level surgery, 8 cases received two-level surgery, 4 cases received three-level surgery, and 1 case received four-level surgery.

**Figure 3:**
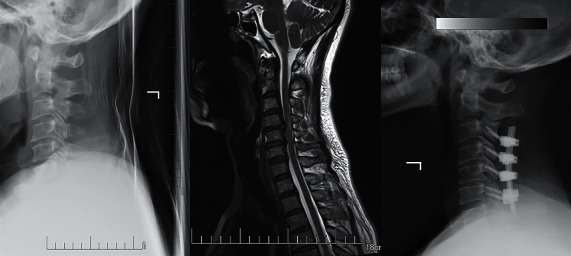
A 65-year-old woman who developed symptoms of ATCCS after a car accident was treated with posterior laminectomy and pedicle screw internal fixation.

**Figure 4:**
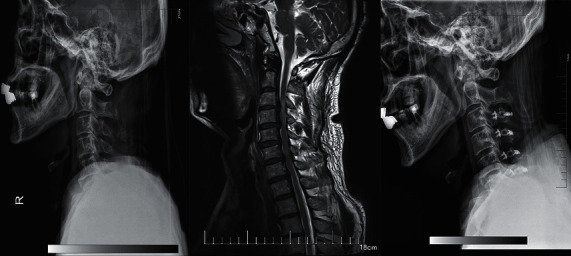
A 61-year-old man who developed symptoms of ATCCS after a fall was treated with posterior expansive open-door laminoplasty.

In Group B, 24 patients received posterior laminoplasty or laminectomy after injury. Of the 11 patients who underwent laminectomy (Figure 3), 2 cases received two-level surgery, 4 cases received three-level surgery, and 5 cases received four-level surgery. Of the 13 patients who underwent laminoplasty (Figure 4), 3 cases received three-level surgery, 6 cases received four-level surgery, and 4 cases received five-level surgery.

### 2.4. Evaluation Indicators

**Figure 5:**
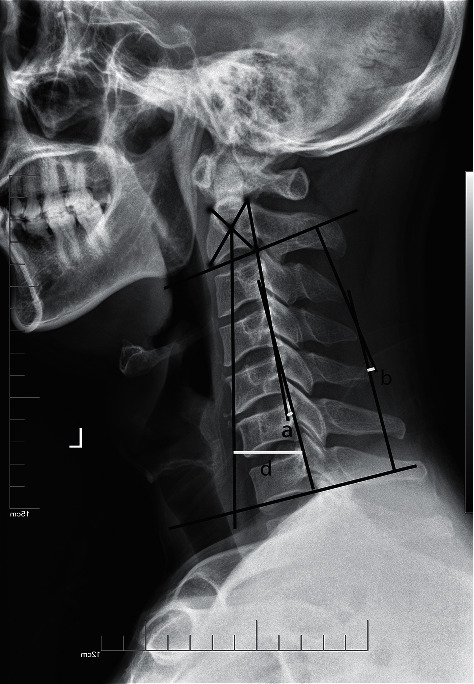
Cervical sagittal parameters. (1) Cervical curvature, Jackson physiological stress curve: two lines are drawn parallel to the posterior edge of C2 and C7; the angle between the two represents the curvature of the cervical spine (a). (2) C2-7 Cobb's angle, the angle between C2 and C7 lower end plate tangent (b). (3) C2–C7 sagittal vertical axis (SVA), the horizontal distance between the back angel of C7 upper end plate and the vertical line of the geometric center of C2 vertebral body (d).

The operation time and intraoperative blood loss, as well as the drainage volume after surgery, were recorded. Spine surgeons also evaluated the visual analogue scale (VAS) score for neck pain of all patients on the first day after surgery and at discharge. Furthermore, some cervical sagittal parameters were recorded to evaluate the postoperative recovery of cervical stability (Figure 5). Spine surgeons mainly measured the following parameters on the lateral cervical X-ray: (1) C2-7 Cobb's angle (the angle between the two represents the curvature of the cervical spine); (2) cervical curvature (Jackson physiological stress curve: draw two lines parallel to the posterior edge of C2 and C7; the angle between the two represents the curvature of the cervical spine); (3) C2–C7 sagittal vertical axis (SVA) (the horizontal distance between the back angel of C7 upper end plate and the vertical line of the geometric center of C2 vertebral body).

Neurological status was assessed using American Spinal Injury Association (Asia) and Japanese Orthopaedic Association (JOA), according to the International Standards for Neurological and Functional Classification of Spinal Cord Injury. In addition, the recovery rate (RR) was calculated based on JOA score.

(1) $$\begin{array}{c} \text{Recovery rate}=\frac{\left(\text{JOA score at discharge/the sixth month}/\text{the final follow}-\text{up}-\text{JOA score at admission}\right)}{\left(17-\text{ JOA score at admission}\right)}\times 100\%. \end{array}$$

As a dependent variable, the JOA score RR at the final follow-up was regarded as an indicator to evaluate the prognosis for patients. The gender, age, causes of injury, time after injury until operation, operation time, blood loss, spinal canal diameter (minimum), CCR, the narrowest segment of the spinal canal, initial Asia grade, and different surgical approaches were added into the risk factor analysis as independent variables.

### 2.5. Statistical Analysis

All data in this study was analyzed using SPSS 26.0 statistical software (SPSS Inc., Chicago, IL). The measurement data was expressed as mean ± standard deviation. Paired sample *T* test was used to compare the two groups. *X*^2^ test was used for categorical variable data. Multiple linear regression was used for correlation analysis (dummy variable assignment was used if the independent variables were unordered multiple categorical variables). *P* < 0.05 indicated that the difference was statistically significant.

## 3. Results

### 3.1. Demographics

Demographic data of both groups are shown in Table 1. Among all patients included in this study, the average age was 57.47 ± 9.70 years. The most common cause of injury was traffic accident, accounting for 40.68%. C4–C5 was the most vulnerable segment of CCS, and the mean CCR was 37.03 ± 8.23%. Patients underwent surgery on an average of 10.88 ± 5.82 days after the injury and were discharged on an average of 9.76 ± 4.65 days after surgery. The average LOS was 15.56 ± 5.84 days, and the average follow-up time was 41.49 ± 14.72 months. There was a 15.25% probability of OPLL among all patients enrolled. Because the anterior approach is usually not chosen for patients with OPLL, there is a significant difference between the two groups in terms of the proportion of OPLL (*P* < 0.05). There were no significant differences between two groups in terms of age, gender, causes of injury, maximal/minimal spinal canal diameter, the narrowest segment of the spinal canal, CCR, time after injury until operation, time after operation until discharge, LOS, comorbidities, and follow-up time (*P* > 0.05).

### 3.2. Secondary Outcome Measures

The results of some secondary outcome measures are shown in Table 2. Although the operation time of Group A was longer than that of Group B (*P* < 0.05), Group B had more intraoperative blood loss and postoperative drainage as well as higher VAS score at discharge compared with Group A (*P* < 0.05). The results of cervical sagittal parameters are shown in Table 3. Although there was no significant difference between the two groups in every period (*P* > 0.05), all parameters in the two groups, including Cobb's angle, cervical curvature, and C2–C7 SVA, improved significantly at discharge, the sixth month after surgery, and the final follow-up compared with the parameters at admission (*P* < 0.05).

### 3.3. Neurological Status Measured Using the American Spinal Injury Association (Asia) Impairment Scale

The results of the Asia grade are shown in Table 4. In the two groups, the Asia grade at discharge, the sixth month after surgery, and the final follow-up showed significant improvement compared with those at admission (*P* < 0.05). At the time of admission, discharge, the sixth month after surgery, and the final follow-up, there was no significant difference in Asia grade between the two groups (*P* > 0.05).

### 3.4. Clinical Function Measured by the Japanese Orthopaedic Association (JOA) Score

The results of the JOA score of patients are shown in Table 5. The JOA score at discharge, the sixth month after surgery, and the final follow-up of the two groups was significantly improved compared with the score at admission (*P* > 0.05). There was no significant difference in JOA score between the two groups at admission, discharge, the sixth month after surgery, and the final follow-up (*P* > 0.05). In addition, the RR of the two groups at the sixth month after surgery and the final follow-up were significantly improved compared to the RR at discharge (*P* < 0.05). However, there was no difference in the RR between two groups at discharge, the sixth month after surgery, and the final follow-up (*P* > 0.05).

Multivariate linear regression analysis of factors was associated with Japanese Orthopaedic Association (JOA) score recovery rate at the final follow-up.

The factors including gender, age, causes of injury, time after injury until operation, operation time, blood loss, spinal canal diameter (minimum), CCR, the narrowest segment of the spinal canal, initial Asia grade, and different surgical approaches were enrolled into multivariate linear regression analysis associated with JOA score RR at the final follow-up. As revealed in Table 6, the results indicated that age, time after injury until operation, and initial Asia grade significantly affected the RR at the final follow-up. Thus, younger age, earlier surgery, and better preoperative Asia grade were positively correlated with better prognosis.

### 3.5. Occurrence of Related Complications

The related complications of patients are shown in Table 7. A total of 5 patients experienced complications during their hospitalization, and there was no statistically significant difference in the incidence of complications between the two groups (*P* > 0.05). Among them, one patient in each of the two groups developed temporary neurological symptoms after surgery, including hoarseness caused by recurrent laryngeal nerve injury in Group A and upper limb weakness caused by C5 nerve root paralysis in Group B, and the two patients all recovered to a good degree through supportive treatment with neurotrophic drugs. In Group A, there was one case of dysphagia and one case of deep vein thrombosis. The patient with dysphagia suffered from pneumonia three days later and was relieved by anti-infection and symptomatic treatments. The patient with deep vein thrombosis was treated using low molecular weight heparin and a pneumatic compression stocking, and this patient did not develop pulmonary embolism. In Group B, there was one patient who suffered from an infection of the surgical wound after surgery. For this patient, third-generation cephalosporin was injected intravenously for 7 days. Eventually, after two weeks of hospitalization, her symptoms disappeared completely.

At the sixth month after surgery, we noticed that 4 patients in Group B reported that they often had varying degrees of axial neck pain, which was significantly different from that in Group A (*P* < 0.05). At the final follow-up, one of the four patients in Group B still had some degree of axial neck pain, but the symptoms of the other three patients almost recovered. In Group A, one patient undergoing ACCF presented with clinical symptoms of adjacent segmental degeneration.

## 4. Discussion

In previous studies, the treatment of patients with ATCCS has been improving in the debate. Until the mid-twentieth century, surgical decompression of SCI had been considered as contraindicated because surgical contusion of the “fragile spinal” cord was suspected to cause further damage [6, 7, 19, 20]. However, with the overall progress of medicine, more and more surgeons advocated that, for patients with spinal instability or continuous compression of the spinal cord, surgical treatments should be recommended [8, 15, 21]. If patients have neurologic deterioration, surgical treatment should be performed in time [22]. In addition, if patients with ATCCS have preexisting multilevel CCS, problems can obviously become more complicated. Previous studies have shown that unless there is only mild spinal cord compression and clinical symptoms, most patients with preexisting multilevel CCS should be treated with surgical decompression, because the spinal cord under continuous compression is difficult to recover in the narrowed spinal canal, which would hinder the recovery of neurological function [10, 14, 23].

In this study, we compared different surgical approaches of the patients with multilevel CCS without cervical fracture or dislocation of ATCCS, in order to determine which surgical approach is preferred for this kind of patients. Due to the lack of specific criteria, the selection of the optimal surgical approach is mainly based on the spinal surgeon's personal experience combined with the patient's imaging results and clinical manifestations [5]. Anterior surgical decompression can stabilize the unstable segment caused by ligament complex injury, but it often requires fusion of more vertebral segments, which may sacrifice more motor segments. Posterior laminoplasty has a wider range of decompression than anterior surgery and can preserve motion segments, but there is often concern about later cervical instability in patients with segmental instability. Posterior laminectomy may provide the desired stability, but it also sacrifices the function of motor segments that require decompression. Therefore, the selection of the optimal surgical approach should be influenced not only by the major compression site of the spinal cord, but also by other factors. The first point is the extent of the pathology. Generally, if the compression is limited to 1–2 levels, the anterior approach is preferred; if more than 2 levels are involved, the posterior approach may be more advantageous [8, 24–27]. The second point to consider is sagittal balance of the cervical spine. The cervical sagittal parameters have been proved to have important reference value for the postoperative recovery of patients undergoing cervical spine surgery and are predictors of clinical outcomes [28]. Harrison et al. [29] conducted a comparative analysis on the changes in C2–C7 Cobb's angle and in cervical curvature and showed that both have high reliability and validity. Chang et al. [30] confirmed that good cervical curvature can keep SVA in a small range. When the cervical curvature is lost, it will make the cervical spine tilt forward and increase SVA. Thus, in this study, we measured Cobb's angle, cervical curvature, and SVA of all patients through cervical X-rays in different periods to compare the effects of different surgical approaches on the sagittal balance of cervical spine in patients with multilevel CCS of ATCCS. No significant difference was found in the results of cervical sagittal parameters between the two groups, which may also be affected by the bias of insufficient follow-up time or the relatively small number of people included in the study.

Few studies have directly discussed which surgical approach is more effective for neurological recovery in patients with preexisting multilevel CCS of ATCCS. In the previous studies, the hot researches mainly focused on the selection of the optimal surgical approach for multilevel cervical spondylotic myelopathy (CSM). Most studies have shown that there was no significant difference in long-term neurological recovery between anterior and posterior approaches in patients with multilevel CSM [31–34]. Furthermore, a study by Brodke et al. [35] compared the efficacy of anterior and posterior surgery in patients with SCI and concluded that there were no significant differences in postoperative pain, complications, and long-term neurological recovery. Our long-term follow-up results of neurological recovery for patients with multilevel CCS of ATCCS are generally consistent with others' conclusions. This may be due to the fact that the ultimate goal of the surgery, whether choosing an anterior or posterior approach, is to allow the compressed spinal cord to have enough space in the narrow spinal canal. Therefore, when surgery can effectively decompress, most patients are able to effectively restore neurological function. However, due to the influence of different surgical approaches, there will inevitably be different results in terms of secondary outcome measures. Anterior cervical spine surgery has the risk of significant complications, such as injury to the thyroid gland, the neurovascular structures of the neck, and injury or rupture of the esophagus, the trachea, or the thoracic duct [36]. Voice changes and dysphagia are common problems after anterior cervical surgery, and recurrent laryngeal nerve injury is the most common nerve injury after an anterior cervical approach [37, 38]. Among patients with the anterior approach in this study, one patient developed dysphagia followed by pneumonia and another patient developed hoarseness due to recurrent laryngeal nerve injury. Posterior cervical spine surgery also brings a risk of serious complications, including C5 nerve root paralysis, axial neck pain, cervical kyphosis, and adjacent segment degeneration [39]. Among patients with posterior approach in this study, one patient had C5 root palsy and four patients had axial neck pain.

Furthermore, the multivariate linear regression analysis revealed that factors significantly associated with JOA score RR at the final follow-up were age, time after injury until surgery, and initial Asia grade. First, the incidence of CCS increases with age, and the degree of spinal cord hyperextension injury in the stenosis spinal canal also increases in these elderly ATCCS [11, 12]. Newey et al. [19] reported poor neurological recovery in patients over 70 years of age. Penrod et al. [40] and Roth et al. [41] also found that age was an adverse prognostic factor associated with functional outcomes. Second, consistent with some studies supporting early surgical decompression [10, 14, 23], early surgical treatment in patients with preexisting multilevel CCS of ATCCS is one of the important factors affecting the long-term recovery of neurological function. Yamazaki et al. [10] and Chen et al. [42] reported that early decompression after injury is one of the positive factors for long-term recovery of neurological function. Third, the Asia score on admission can truly predict the long-term neurological recovery of patients to a certain extent, because the Asia score is an objective indicator of neurological impairment [41, 43].

It is well known that MP plays a critical role in the early management of patients with acute SCI. According to the latest guidelines of AOSpine [44], 24-hour MP shock therapy is recommended for adult patients with acute SCI who are injured within 8 hours, but 48-hour high-dose MP infusion is not suggested. Therefore, we included all patients in this study following the standard treatment regimen, i.e., 24-hour MP shock therapy within 8 hours of injury, which made the comparison of long-term outcomes between the two groups more convincing.

This study had several limitations. First, it was designed as a retrospective comparative study, and the sample size was relatively insufficient. Second, there were no specific criteria for the indication of anterior or posterior surgery, because the experience of the spine surgeon was the dominant factor in most cases. Third, there are two approaches to both the anterior and posterior surgeries, and we did not further distinguish them to study, which would affect the comparison of secondary outcome measures and the recovery of neurological function to a certain extent. Therefore, prospective controlled studies are needed to study more patients and make more in-depth comparison of anterior and posterior surgery.

## 5. Conclusions

For patients with multilevel CCS without cervical fracture or dislocation of ATCCS, both surgical approaches can achieve good outcomes. During follow-up, there was no significant difference in the neurological function recovery between the two groups. Although no significant differences were found in the primary outcome measures, some secondary outcome measures favored the anterior approach and others favored the posterior approach. Younger age, earlier surgery, and better preoperative Asia grade were protective factors for better prognosis.

## Figures and Tables

**Table 1:** Demographics of the two groups.

	Full sample (*N* = 59)	Group A (*n* = 35)	Group B (*n* = 24)	*P* value
Age (years)	57.47 ± 9.70	56.11 ± 9.29	59.46 ± 10.14	0.126
Gender (male/female)	38/21	24/11	14/10	0.420
Causes (*n*)				0.867
Traffic accident	24 (40.68%)	14 (40.00%)	10 (41.67%)	
Falling	20 (33.90%)	12 (34.29%)	8 (33.33%)	
Sports	8 (13.56%)	4 (11.43%)	4 (16.67%)	
Others	7 (11.86%)	5 (14.28%)	2 (8.33%)	
Spinal canal diameter (mm)				
Minimum	6.60 ± 1.05	6.54 ± 1.05	6.68 ± 1.56	0.540
Maximum	10.45 ± 0.70	10.43 ± 0.72	10.48 ± 0.68	0.711
Canal compression rate (%)	37.03 ± 8.23	37.43 ± 8.27	36.45 ± 8.23	0.592
The narrowest segment of the spinal canal (*n*)				0.723
C2-3	0 (0%)	0 (0%)	0 (0%)	
C3-4	10 (16.95%)	6 (17.14%)	4 (16.67%)	
C4-5	31 (54.54%)	18 (51.43%)	13 (54.16%)	
C5-6	13 (22.03%)	9 (25.71%)	4 (16.67%)	
C6-7	5 (8.48%)	2 (5.72%)	3 (12.50%)	
Time after injury until operation (days)	10.88 ± 5.82	10.14 ± 6.34	11.96 ± 4.85	0.143
Time after operation until discharge (days)	9.76 ± 4.65	9.11 ± 4.70	10.71 ± 4.46	0.120
OPLL (*n*)	9 (15.25%)	1 (2.86%)	8 (33.33%)	<0.001^*∗*^
LOS (days)	15.56 ± 5.84	14.86 ± 5.98	16.58 ± 5.53	0.178
Comorbidities (*n*)				
Hypertension	21 (35.59%)	12 (34.29%)	9 (37.50%)	0.800
Diabetes	19 (32.20%)	11 (31.43%)	8 (33.33%)	0.878
Hyperlipidemia	15 (25.42%)	9 (25.71%)	6 (25.00%)	0.951
Smoking	16 (27.12%)	9 (25.71%)	7 (29.17%)	0.770
Follow-up period (months)	41.49 ± 14.72	40.80 ± 13.68	42.29 ± 16.40	0.706

LOS: length of stay; OPLL: ossification of the posterior longitudinal ligament. ^*∗*^Significance between the two groups, *P* < 0.05.

**Table 2:** Secondary outcome measures.

	Full sample (*N* = 59)	Group A (*n* = 35)	Group B (*n* = 24)	*P* value
Operation time (minutes)	197.15 ± 30.93	214.54 ± 21.87	171.79 ± 23.96	<0.001^*∗*^
Blood loss (ml)	368.22 ± 120.09	336.71 ± 125.77	414.17 ± 96.27	0.014^*∗*^
Drainage (ml)	96.53 ± 37.02	79.54 ± 30.16	121.29 ± 32.14	<0.001^*∗*^
VAS scores				
Postoperative day 1	3.81 ± 0.84	3.80 ± 0.93	3.83 ± 0.70	0.883
Discharge	1.17 ± 0.85	0.91 ± 0.70	1.54 ± 0.93	0.005^*∗*^

VAS: visual analogue scale. ^*∗*^Significance between the two groups, *P* < 0.05.

**Table 3:** Cervical sagittal parameters.

	Group A (*n* = 35)	Group B (*n* = 24)	*P* value
C2–C7 Cobb's angle (°)			
Admission	12.89 ± 2.10	13.71 ± 1.78	0.122
Discharge	16.86 ± 2.40^*∗*^	17.17 ± 2.14^*∗*^	0.614
Sixth month	16.37 ± 2.66^*∗*^	16.21 ± 2.45^*∗*^	0.812
Final visit	16.09 ± 2.45^*∗*^	15.67 ± 2.35^*∗*^	0.515

Cervical curvature (°)			
Admission	12.83 ± 1.56	13.13 ± 1.89	0.513
Discharge	15.69 ± 2.27^*∗*^	15.25 ± 1.96^*∗*^	0.448
Sixth month	15.34 ± 2.82^*∗*^	15.00 ± 1.84^*∗*^	0.603
Final visit	15.03 ± 2.65^*∗*^	14.58 ± 1.95^*∗*^	0.486

C2–C7 SVA (mm)			
Admission	28.00 ± 6.43	29.33 ± 7.39	0.464
Discharge	20.86 ± 4.19^*∗*^	21.04 ± 4.28^*∗*^	0.870
Sixth month	21.69 ± 3.76^*∗*^	22.67 ± 4.27^*∗*^	0.355
Final visit	23.11 ± 5.19^*∗*^	24.00 ± 6.74^*∗*^	0.571

SVA: sagittal vertical axis. ^*∗*^Significance compared with the value at admission, *P* < 0.05.

**Table 4:** Neurological status measured by the American Spinal Injury Association (Asia) grade.

Group (*n* = 59)	Admission				Discharge					Sixth month					Final visit				
	B	C	D	E	B	C	D	E		B	C	D	E		B	C	D	E	
A (35)	2	10	23	0	0	4	19	12	^*∗*^	0	0	12	23	^*∗*^	0	0	9	26	^*∗*^
B (24)	5	7	12	0	0	7	11	6	^*∗*^	0	3	7	14	^*∗*^	0	1	9	14	^*∗*^

^*∗*^Significance compared with the Asia grade at admission, *P* < 0.05.

**Table 5:** Clinical function measured by the Japanese Orthopaedic Association (JOA) score.

Group (*n* = 59)	Admission	Discharge		Sixth month		Final visit	
	JOA score	JOA score	RR (%)	JOA score	RR (%)	JOA score	RR (%)
A (35)	9.40 ± 2.37	11.60 ± 2.15^*∗*^	30.23 ± 10.80	13.91 ± 1.42^*∗*^	60.07 ± 10.36^†^	14.77 ± 1.52^*∗*^	72.97 ± 13.03^†^
B (24)	8.63 ± 2.58	10.92 ± 2.25^*∗*^	27.93 ± 10.52	13.58 ± 1.47^*∗*^	59.70 ± 10.13^†^	14.33 ± 1.74^*∗*^	70.86 ± 12.80^†^

JOA: Japanese Orthopaedic Association; RR: recovery rate. ^*∗*^Significance compared with the JOA score at admission, *P* < 0.05; ^†^significance compared with the RR at discharge, *P* < 0.05.

**Table 6:** Multivariate linear regression analysis of factors associated with Japanese Orthopaedic Association score recovery rate at the final follow-up.

Parameters	Standardized coefficient	SE	*P* value
Gender	0.068	0.028	0.523
Age	−0.208	0.001	0.049^*∗*^
Causes of injury	0.121	0.014	0.240
Time after injury until operation	−0.308	0.002	0.009^*∗*^
Operation time	−0.055	0.001	0.707
Blood loss	−0.050	0.000	0.670
Spinal canal diameter (minimum)	−0.333	0.031	0.194
Canal compression rate	−0.124	0.377	0.608
The narrowest segment of the spinal canal	0.014	0.016	0.894
Initial ASIA grade	0.595	0.022	<0.001^*∗*^
Surgical approach	0.077	0.035	0.571

^*∗*^represents statistically significant, *P* < 0.05.

**Table 7:** Postoperative complications.

	Group A (*n* = 35)	Group B (*n* = 24)	*P* value
During hospitalization	Dysphagia and pneumonia (1)	Surgical wound infection (1)	0.974
	Deep vein thrombosis (1)	C5 nerve root paralysis (1)	
	Recurrent laryngeal nerve injury (1)		
Sixth month	—	Axial neck pain (4)	0.012^*∗*^
Final visit	Adjacent segment degeneration (1)	Axial neck pain (1)	0.789

^*∗*^Significance between the two groups, *P* < 0.05.

## Data Availability

Data are available upon request to the corresponding author.

## Abbreviations

CCS: Cervical canal stenosis
ATCCS: Acute traumatic central cord syndrome
Asia: American Spinal Injury Association
JOA: Japanese Orthopaedic Association
RR: Recovery rate
SCI: Spinal cord injury
ACCF: Anterior cervical corpectomy and fusion
ACDF: Anterior cervical discectomy and fusion
VAS: Visual analogue scale
CCR: Canal compression rate
LOS: Length of stay
OPLL: Ossification of the posterior longitudinal ligament
SVA: Sagittal vertical axis
CSM: Cervical spondylotic myelopathy
MP: Methylprednisolone.
